# Role of PTEN in Oxidative Stress and DNA Damage in the Liver of Whole-Body Pten Haplodeficient Mice

**DOI:** 10.1371/journal.pone.0166956

**Published:** 2016-11-28

**Authors:** Ezgi Eyluel Bankoglu, Oliver Tschopp, Johannes Schmitt, Philipp Burkard, Daniel Jahn, Andreas Geier, Helga Stopper

**Affiliations:** 1 Institute of Pharmacology and Toxicology, University of Wuerzburg, Wuerzburg, Germany; 2 Clinic for Endocrinology & Diabetology, University Hospital Zuerich, Zuerich, Switzerland; 3 Division of Hepatology, Department of Medicine II, University Hospital Wuerzburg, Wuerzburg, Germany; North Carolina State University, UNITED STATES

## Abstract

Type 2 diabetes (T2DM) and obesity are frequently associated with non-alcoholic fatty liver disease (NAFLD) and with an elevated cancer incidence. The molecular mechanisms of carcinogenesis in this context are only partially understood. High blood insulin levels are typical in early T2DM and excessive insulin can cause elevated reactive oxygen species (ROS) production and genomic instability. ROS are important for various cellular functions in signaling and host defense. However, elevated ROS formation is thought to be involved in cancer induction. In the molecular events from insulin receptor binding to genomic damage, some signaling steps have been identified, pointing at the PI3K/AKT pathway. For further elucidation Phosphatase and Tensin homolog (Pten), a tumour suppressor phosphatase that plays a role in insulin signaling by negative regulation of PI3K/AKT and its downstream targets, was investigated here. Dihydroethidium (DHE) staining was used to detect ROS formation in immortalized human hepatocytes. Comet assay and micronucleus test were performed to investigate genomic damage *in vitro*. In liver samples, DHE staining and western blot detection of HSP70 and HO-1 were performed to evaluate oxidative stress response. DNA double strand breaks (DSBs) were detected by immunohistostaining. Inhibition of PTEN with the pharmacologic inhibitor VO-OHpic resulted in increased ROS production and genomic damage in a liver cell line. Knockdown of Pten in a mouse model yielded increased oxidative stress levels, detected by ROS levels and expression of the two stress-proteins HSP70 and HO-1 and elevated genomic damage in the liver, which was significant in mice fed with a high fat diet. We conclude that PTEN is involved in oxidative stress and genomic damage induction in vitro and that this may also explain the in vivo observations. This further supports the hypothesis that the PI3K/AKT pathway is responsible for damaging effects of high levels of insulin.

## 1 Introduction

Obesity is closely associated with insulin resistance, hyperinsulinemia and type 2 diabetes mellitus (T2DM) and the parallel increase in the prevalence of obesity and T2DM is a rising health concern [1, 2]. Epidemiological studies have assessed the association between obesity/T2DM and cancer in large populations and documented positive trends in death-rates with increasing body-mass-index for various cancers including liver [3–5]. Non-alcoholic fatty liver disease (NAFLD) frequently occurs in the context of obesity, insulin resistance and T2DM and is increasingly recognized in industrialized countries worldwide [6]. Although the association of NAFLD with insulin resistance and T2DM is well established, the molecular mechanisms of carcinogenesis are still only partially understood.

High blood insulin levels are typical before manifestation and in the early years of T2DM [7, 8]. Excessive insulin can cause mitochondrial dysfunction and overactivation of NADPH oxidase [9]. In both cases, it results in elevated reactive oxygen species (ROS) production. ROS are required for various cellular functions in signalling and host defense. However, excessive ROS formation can lead to cellular alterations and damage [10]. If the antioxidant defense is overwhelmed, elevated oxidative stress can cause DNA damage and increase the risk of various cancer types [11]. ROS production, stress responses and therefore genomic stability can be affected by life style factors [12, 13].

In the series of molecular events leading from insulin receptor binding to genomic damage, some signaling steps have been elucidated, pointing at the PI3 kinase/AKT pathway [14]. One important enzyme complex that may contribute to this pathway is Phosphatase and Tensin homolog (PTEN), a tumour suppressor phosphatase that plays a key role in insulin signaling by dephosphorylation of the phosphoinositides and negative regulation of PI3K and its downstream targets [15]. Indeed, mice with a hepatocyte-specific deletion of Pten show age-dependent development of liver steatosis and hepatocellular carcinoma [16, 17]. In humans, Pten mutations have been described as a cause of constitutive insulin sensitivity and obesity [18]. In fatty liver patients, reduced expression of PTEN and concomitant hyperactivation of AKT have been observed in liver biopsy tissue [19]. There is particularly strong evidence of obesity-related disorders as risk factors for hepatocellular carcinoma and of NAFLD as a cause of hepatocellular carcinoma [20].

However, further elucidation of the role of obesity, insulin signaling and the particular role of PTEN in cancer development is still required. Therefore we investigated the role of PTEN in the production ROS and genomic damage in a hepatocyte cell line *in vitro* and in Pten haplodeficient mice.

## 2 Materials and methods

### 2.1 Chemicals, reagents and antibodies

William`s Medium E (W1878), Dexamethasone (D4902) and fluorescein diacetate (FDA) were obtained from Sigma Aldrich (Steinheim, Germany or St. Louis, USA). Other cell culture reagents were obtained from PAA Laboratories GmbH (Pasching, Austria) and Invitrogen Life Technologies (Carlbad, USA). Fetal bovine serum (FBS) was from Biochrom (Berlin, Germany). Human insulin solution was purchased from Santa Cruz Biotechnology (Santa Cruz, CA, USA). PTEN inhibitor (VO-OHpic, Trihydrate) was obtained from BioVison, Inc. (Milpitas, CA, USA). Dihydroethidium (DHE) was purchased from Merck Biosciences GmbH (Schwalbach, Germany). Gel Red and Gel Green were purchased from Biotrend (Köln, Germany). Normal donkey serum was obtained from Milipore (Temecula, USA). Peroxidase Sunstrate kit (SK-4100) and Vectastain ABC kit (PK-6100) were purchased from Vector Laboratories, Inc. (Burlingame, USA). Gel Red was from Biotium (Hayward, CA, USA). The antibodies against beta-actin (3700s), p-Histone H2A.X (S139) (20E3), PTEN (138G6), pan-AKT (C67E7), pAKT S473 (D9E) and pAKT T308 (D25E6) were obtained from Cell Signalling Technology Inc (Beverly, USA). The antibody against HSP70 (sc-24) was obtained from Santa Cruz Biotechnology (Santa Cruz, CA, USA) and HO-1 (ab68477) was obtained from Abcam (Cambridge, UK) (The secondary antibodies HRP conjugated goat anti-mouse IgG (sc-2005) and biotin conjugated antibody donkey anti-rabbit IgG-B (sc-2089) were purchased from Santa Cruz Biotechnology (Santa Cruz, CA, USA). The secondary antibody HRP conjugated anti-rabbit IgG (7074s) was purchased from Cell Signalling Technology Inc (Beverly, USA). TRIZOL and SYBR Green were obtained from Invitrogen (Carlsbad, CA).

### 2.2 Cell culture and treatment of cells

The immortalized human hepatocyte cell line IHH was cultured in William`s Medium E with 10% (v/v) fetal bovine serum, 1% (w/v) L-glutamine, 1% (w/v) antibiotic (50 U/ml penicillin and 50 mg/ml streptomycin), 1 mU/ml human insulin and 50 nM/L dexamethasone in an incubator with 5% CO_2_, 37°C. IHH cells were supplied from Prof. Dr. Folkert Kuipers (Department of Pediatrics, University Hospital Groningen, Groningen, Netherlands).

One day prior to experiments, immortalized human hepatocyte (IHH) cells [21] were inoculated in 6 well plates containing 3 ml medium per well. On the day of the experiment, cells were placed in medium, which did not contain insulin and dexamethasone and were pre-incubated with a vanadium complex that is a specific inhibitor of PTEN (VO-OHpic, 50nM) for 15 min at 37°C. After 15 min pre-incubation, cells were treated for an additional 30 min (for DHE staining), 2h (for comet assay) or 4h (for micronucleus test) with insulin. HCl solution (0.05 M) was used to dilute insulin and as negative control.

### 2.3 Vitality test

Toxicity of treatments was tested by a dye exclusion/activation fluorescent vitality test. For this, 70 μl of cell suspension was mixed with 30 μl of staining solution (2μl Gel Red stock solution and 12μl fluorescein diacetate (FDA; 5mg/ml in acetone) in 2 ml PBS. Twenty μl of this mixture was applied on the slide and covered with a cover slip (21x26 mm). In total 200 cells (red and green stained) were counted at 200-fold magnification with an Eclipse 55i microscope which was equipped with FITC filter (Nikon GmbH, Düsseldorf, Germany). The proportion of green cells (vital) to red cells (dead) was evaluated.

### 2.4 Microscopic detection of intracellular ROS

The cell permeable fluorescent dye dihydroethidium (DHE) was used to detect intracellular ROS. DHE (5 μM) was added during the 30 min incubation with insulin (after pretreatment with the Pten inhibitor) at 37°C in dark. At the end of the incubation time, cells were washed with PBS and pictures of the cells were taken with an Eclipse 55i microscope (Nikon GmbH, Düsseldorf, Germany) and a Fluoro Pro MP 5000 camera (Intas Science Imaging Instruments GmbH, Göttingen, Germany) at 200-fold magnification. Two hundred and fifty cells were analysed per group by using ImageJ software (http://rsbweb.nih.gov/ij).

### 2.5 Alkaline comet assay

The alkaline comet assay is a single gel electrophoresis assay, which can detect single and double strand breaks and alkali labile sites. After treatment of the cells with 50 nM VO-OHpic for 15 min, insulin was added for an additional two hours. Then, cells were harvested and 20 μl of cell suspension was mixed with 180 μl of 0.5% low melting point agarose at 37°C. Forty-five μl of cell-agarose mixture was placed on a fully frosted slide that was coated with 1.5% of high melting point agarose. The cell suspension was covered with a cover glass and the slides were kept at 4°C for 30 sec to solidify the mixture. After gentle removal of the cover-glass, slides were kept in a lysis solution (1% Triton X-100, 10% dimethyl sulfoxide, and 89% lysis buffer containing 10mM Tris, pH 10; 1% Na-sarcosine; 2.5M NaCl; and 100mM Na_2_EDTA) for 1 hour in dark at 4°C. After the lysis step, to allow DNA unwinding, the slides were placed in an electrophoresis chamber that was filled with fresh electrophoresis buffer (300mM NaOH and 1mM Na_2_EDTA, pH 13) for 20 min at 4°C in dark. Then the electrophoresis was performed for 20 min at 25 V (1.1 V/ cm) and 300 mA. To neutralize the slides they were immersed in 0.4 M Tris buffer (pH 7.5) for 5 min and then to dehydrate in methanol for 5 min at −20°C. The slides were left to dry under a fume hood and stained with 20 μl of Gel red/ Dabco solution. Evaluations of the slides were done with a fluorescence microscope (Labophot 2; Nikon) at 200-fold magnification and image analysis software (Komet 5, BFI Optilas, Germany). One hundred randomly selected cells (50 per replicate slide) for each treatment were analyzed. The percentage of DNA in the tail was used to quantify DNA damage.

### 2.6 Cytokinesis-block micronucleus assay

Micronucleus formation is a marker of genomic damage on the chromosomal level. A micronucleus forms during mitosis due to the chromosome breakage or whole chromosome maldistribution. IHH cells were pre-incubated with 50 nM of VO-OHpic for 15 min at 37°C. Afterwards insulin was added for an additional 4 hours. Following to treatment of IHH cells, the medium was removed and replaced by fresh culture medium after washing with PBS. IHH cells were incubated with 3 μg/mL cytochalasin B for 24 hours to block separation of daughter cells yielding cells with two daughter nuclei, i.e. binucleated cells. After cytokinesis-block, cells were harvested, placed on a glass slide by cytospin centrifugation and fixed in methanol at – 20°C for at least 2 hours. Before counting, cells were stained with gel green (1:100 diluted in bidistilled water) and mounted with Dabco for microscopy. Micronuclei were counted with an Eclipse 55i microscope (Nikon GmbH, Düsseldorf, Germany) at 400-fold magnification by using a FITC filter. For each treatment, two replicate slides were prepared and 1000 binucleated cells (BN) per slide were scored for micronuclei. In addition, 1000 cells were scored for mononucleated cells, binucleated cells, multinucleated cells, mitosis and apoptosis. The cytokinesis block proliferation index (CBPI) as an indicator of cytostatic effects was determined by using the equation below:

$$\frac{\left(number\;of\;mononucleated\;cells+2xnumber\;of\;binucleated\;cells+3xnumber\;of\;multinucleated\;cells\right)}{\left(sum\;of\;mononucleated,\;binucleated\;and\;multinucleated\;cells\right)}$$

### 2.7 Animal model

Mice with whole-body targeted deletion of *Pten* were described previously and were in a C57BL/6 background after at least 6 backcrosses [22, 23]. The heredity of targeted alleles was confirmed by PCR. Mice were housed according to the Swiss Animal Protection Laws in groups with a 12-h dark-light cycle and free access to food and water, unless otherwise indicated. Starting at an age of 12 weeks, mice were fed with normal diet (ND; Kliba 3336 containing 5.5% crude fat) or high fat diet (HFD; Kliba 2126 containing 23.6% crude fat) for a duration of 20 weeks. All animal experiments were performed in accordance with Swiss Federal Animal Regulation and approved by the Veterinary Office of Zurich, Switzerland (No: 35/2009 and 222/2012). Mice were terminally euthanized using carbon dioxide.

### 2.8 Enzymatic determination of hepatic triacylglyceride

To measure hepatic triacylglyceride content, lipids were extracted from approximately 30 mg of liver tissue by homogenization in 500 μl acetone. Homegenates were centrifuged twice for 5 min at full speed to remove the cell debris. The hepatic triacylglyceride concentrations were measured by using TRIG kits (Diatools, Villmergen, Switzerland) according to the manufacturer`s introductions.

### 2.9 Quantitative real time PCR

Total RNA was isolated from liver tissue using TRIZOL (Invitrogen, Carlsbad, CA) following the manufacturer’s instructions. cDNA synthesis was performed with M-MuLV reverse transcriptase (New England BioLabs, Ipswich, MA, USA) according to the manufacturer’s instructions. Quantitative real time PCR reactions were performed using SYBR Green (Invitrogen, Carlsbad, CA) on ABI Prism 7000 or StepOnePlus Real-Time PCR System (Applied Biosystems, Rotkreuz, Switzerland). Primer sequence was obtained from PrimerBank [24]. The primer used and the corresponding PrimerBank ID was Fas (ID: 30911099a2).

### 2.10 Microscopic detection of ROS on organ slices

Cryosections from snap-frozen liver (5μm) tissues were prepared with a Leica CM3050 Scryostat (Leica, Wetzlar, Germany). The sections were stained with 10 μM DHE for 30 min in the dark at room temperature. After staining, sections were washed with PBS, mounted and cover with a cover glass. The pictures of the cells were taken with an Eclipse 55i microscope (Nikon GmbH, Düsseldorf, Germany) and a Fluoro Pro MP 5000 camera (Intas Science Imaging Instruments GmbH, Göttingen, Germany) at 400-fold magnification. Quantification was done by measuring grey values of 200 cells per animal with ImageJ 1.40g (http://rsb.info.nih.gov/ij/).

### 2.11 Western Blot analysis

Part of snap-frozen liver tissue was homogenized by using an ultra-turrax in ice-cold radio immune-precipitation assay (RIPA) buffer, which contained freshly added protease inhibitor cocktail (PIC), sodium orthovanadate and sodium fluoride to inhibit protease and phosphatase activity. After homogenization of tissues, samples were centrifuged at 14,000 rpm and 4°C for 15 min. The protein lysate was stored at -20°C until the performance of western blot analysis. The protein concentration of samples were determined by using Bio-Rad protein assay (based on Bradford`s method). Fifty μg of protein per sample was loaded on SDS gel and electrophoresis was performed in order to separate proteins. The separated proteins were transferred from gel to PVDF membrane (polyvinylidene difluoride membranes, 0.2 μ). The membrane was blocked at least for 2 hours in 5% of skim milk powder in TBS-T buffer (5 mM TRIS, 150 mM NaCl, 0.05% (w/v) Tween-20). After blocking, membrane was incubated with primary antibody [heat shock protein 70 (HSP70, 1:1000); heme oxygenase-1 (HO-1, 1:5000); phosphatase and tesin homolog (PTEN, 1:5000); pan-AKT (1:5000); pAKT S473 (1:5000); pAKT T308 (1:5000) and β-actin (1:5000)] overnight at 4°C. At the next day, the membranes were incubated with horseradish peroxidase (HRP) conjugated secondary antibody (1:5000) for an hour and next with HRP substrate for 5 min. The membrane was exposed to an x-ray sensitive film and the film was developed later on. The quantification of bands were performed by Image j software (http://rsbweb.nih.gov/ij). Results were normalized to the endogenous control β-actin.

### 2.12 Detection of phosphorylated H2AX sites

Antibody staining against phosphorylated Ser-139 at the C-terminus of histone H2AX was performed on paraffin sections of the liver samples to detect double strand breaks (DSBs). The paraffin sections (2 μm) were prepared with a microtome (LEICA RM 2165, Wetzlar, Germany) and mounted on positively charged slides. The sections were heated at 60°C for an hour, deparaffinised (3 x 4 min Roti-Histol, 2 x 3 min 100% EtOH, 1 x 2 min 70% EtOH) and washed with PBS. Antigen retrieval was achieved by cooking the slides for 4 min in citrate buffer (10 mM sodium citrate, pH 6) on the highest level in a pressure cooker. To cool down, the slides were washed with PBS. The sections were blocked in 5% donkey serum for an hour at room temperature in dark. To reduce background staining, endogenous peroxidase activity was depleted by incubation with 3% of H_2_O_2_ for 15 min at room temperature in dark. Prior to the incubation of biotinylated antibody, slides were incubated successively with 0.001% avidin and biotin for 15 min at room temperature in the dark. Later on, primary antibody (phospho-histone H2AX (Ser139, clone 20E3) rabbit mAb; 1:200) incubation was performed overnight at 4°C in the dark. Following the washing steps, biotin-conjugated secondary antibody (donkey anti rabbit IgGB, sc2089, Santa Cruz Biotechnology; 1:200) incubation was performed for 45 min, at room temperature in dark. After washing the sections with PBS, the sections were incubated with the avidin biotin complex (ABC) reagent (Vectastin-Elite ABC reagent: two drops of reagent A and reagent B mixed with 10 mM sodium phosphate, 0.9% NaCl, pH 7.5) for 30 min at room temperature in dark and washed again in PBS. Sections were incubated with diaminobenzidin (DAB) chromogen (Vector Laboratories) for 10 min at room temperature. DAB reacts with HRP in the presence of peroxide to yield an insoluble brown-colored product at locations where peroxidase-conjugated antibodies are bound to samples. Counterstaining was done with Ehrlich’s hematoxylin (1 g hematoxylin, 48 mL 99.8% isopropanol, 51.9 mL H2O d, 50 mL glycerol, 1.5 g potassium alum, 5 mL acetic acid, 0.2 g potassium iodate) for three minutes and then the sections were washed for 10 min in pure water. Before mounting with Eukitt (Fluka), the sections were dehydrated (1 x 1 min 70% EtOH, 4 x 2 min 100% EtOH, 2 x 3 min Roti-Histol). Pictures were taken with a Keyence BZ-9000 microscope at 400-fold magnification. The percentage of positive cells was assessed by scoring 15 to 20 images per sample.

### 2.13 Statistical analysis

Data are presented as mean ± SEM. Statistical analysis was performed with SPSS 22 software. Kruskal-Wallis test was used to determine significance between multiple groups and the Mann-Whitney U-test was used to determine the significance between two groups. Results with p values of ≤ 0.05 were considered as significant.

## 3 Results

### 3.1 Modulation of insulin-induced oxidative stress and genomic damage in liver cells in vitro

#### 3.1.1 Intracellular ROS in IHH cells

**Fig 1:**
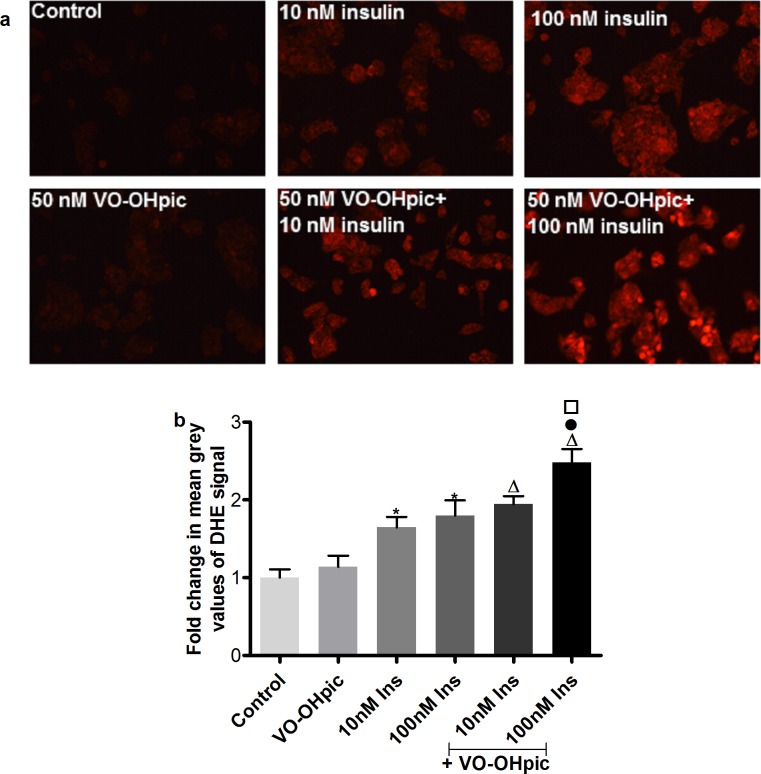
Intracellular oxidative stress induction in IHH cells by insulin treatment with or without the PTEN inhibition. **a.** Representative pictures for DHE staining. **b.** Quantification of DHE fluorescence by measuring the mean grey value of DHE signal from 250 cells using image j software. Data are presented as mean fold change compared to control ± SEM of 5 independent experiments. * p≤0.05 vs. solvent control, Δ p≤0.05 vs. VO-OHpic, ● p≤0.05 vs. 100 nM insulin and □ p≤0.05 vs. VO-OHpic + 10 nM insulin.

DHE staining was applied to monitor intracellular ROS after treating the cells with 10 and 100 nM insulin for 30 min with and without the addition of the PTEN inhibitor VO-OHpic (50nM). As shown in **Fig 1A**, insulin treatment caused an increase in red color intensity. The intensity was further enhanced by addition of the PTEN inhibitor. Quantification of the staining intensity is shown in **Fig 1B**. Treatment with 10 and 100 nM insulin caused significant induction of ROS. The combination of the PTEN inhibitor with 100 nM insulin yielded a significant increase in ROS formation compared to the 100 nM insulin treated cells.

### 3.1.2 Genomic damage in IHH cells

Two different methods were used to monitor the modulation of the genotoxic potential of insulin in IHH cells, the alkaline comet assay and the cytokinesis block micronucleus test.

**Fig 2:**
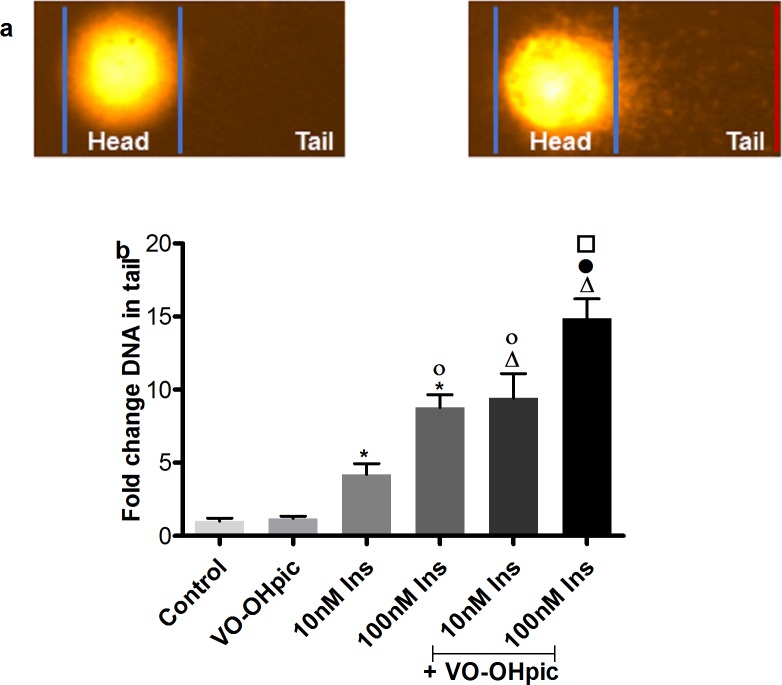
DNA damage induction in IHH cells by insulin treatment with or without PTEN inhibition. **a.** Representative pictures for comets of the control (left) and of insulin-treated (right) IHH cells. **b.** Quantification of comet assay results. Data are presented as mean fold change compared to control ± SEM of 4 independent experiments. * p≤0.05 vs. HCl, Δ p≤0.05 vs. VO-OHpic, ○ p≤0.05 vs. 10 nM insulin, ● p≤0.05 vs. 100 nM insulin and □ p≤0.05 vs. VO-OHpic + 10 nM insulin.

The alkaline comet assay was used to show single and double strand breaks or alkali-labile sites of DNA. A typical comet picture can be seen in **Fig 2A**. In the alkaline comet assay, IHH cells were treated for 2 hours with insulin with or without the presence of the PTEN inhibitor VO-OHpic. As displayed in **Fig 2B**, insulin treatment caused a dose dependent and significant induction of DNA damage. The PTEN inhibition induced a further increase of DNA damage. There was no significance difference in cell viability (**Table 1**).

**Table 1:** Viability of cells after treatment with insulin and the PTEN inhibitor VO-OHpic.

Treatment Groups	Viability (%)
**Control**	93.88 ± 1.60
**VO-OHpic**	96.88 ± 0.83
**10nM Insulin**	97.38 ± 0.75
**100nM Insulin**	96.13 ± 1.53
**VO-OHpic + 10 nM Insulin**	96.88 ± 1.39
**VO-OHpic + 100nM Insulin**	94.38 ± 1.13

Date are shown as mean ± SEM from 4 independent experiments.

**Fig 3:**
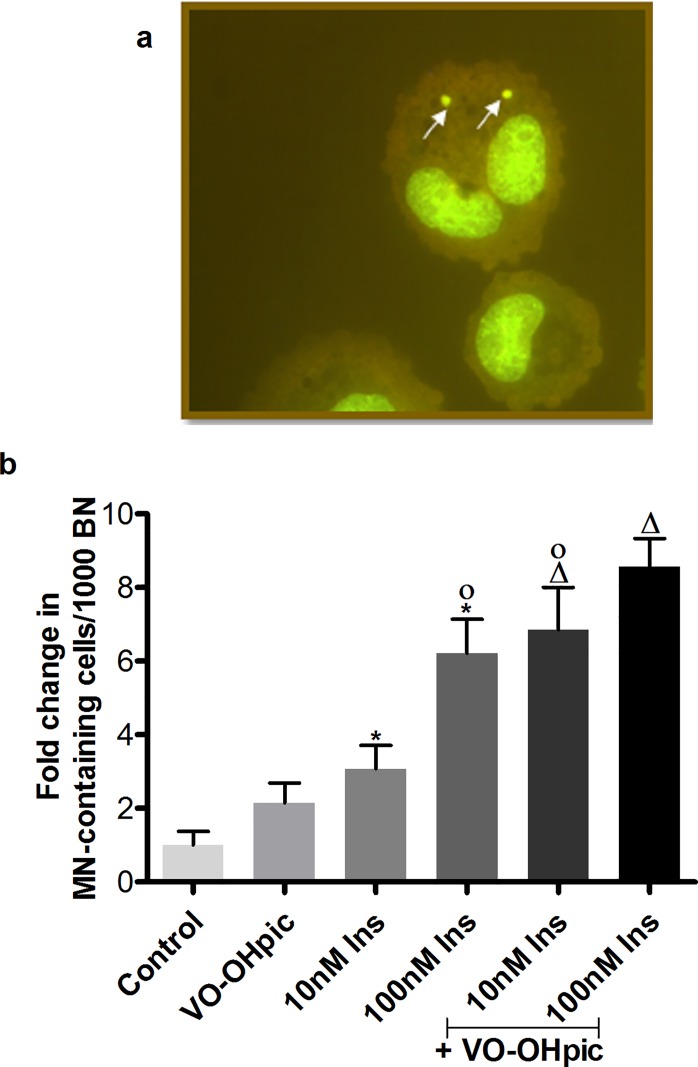
MN induction in IHH cells by insulin treatment with or without PTEN inhibition. **a.** Representative picture of micronuclei in a binucleated cell. **b.** Quantification of micronucleus test. Data are presented as mean fold change of MN-containing cells in 1000 binucleated cells (BN) compared to control ± SEM of 4 independent experiments. * p≤0.05 vs. solvent control, Δ p≤0.05 vs. VO-OHpic, ○ p≤0.05 vs. 10 nM insulin, ● p≤0.05 vs. 100 nM insulin. ▫ p≤0.05 vs. VO-OHpic + 10 nM insulin and ▪ p≤0.05 vs. VO-OHpic + 100 nM insulin.

The cytokinesis block micronucleus test was used to monitor irreversible chromosomal damage in vitro. A typical appearance of micronuclei in a binucleated cell (BNC) can be seen in **Fig 3A** and the quantification of the cytokinesis block micronucleus test for IHH cells is shown in **Fig 3B**. After 4 hours of treatment with 10 and 100 nM of insulin, a significant induction of micronuclei (MN) formation was observed. The combination of the PTEN inhibitor VO-OHpic with 10 and 100 nM insulin yielded a significantly elevated induction in micronucleus formation compare to the VO-OHpic treated group as well as compared with insulin alone, which was significant for 10nM insulin. Additionally, apoptotic and mitotic cells were counted (**Table 2**). Insulin, especially at 10nM, provided a non-significant trend for protection from apoptosis and increased mitotic activity, but none of our treatment conditions induced any significant effect on apoptosis and mitosis. There was no significant increase in cytokinesis block proliferation index (CBPI), which can be seen in **Table 3**.

**Table 2:** Apoptotic and mitotic cells after treatment with insulin and the Pten-inhibitor VO-OHpic.

Treatment Groups	Apoptosis	Mitosis
**Control**	1 ± 0.23	1 ± 0.1
**VO-OHpic**	0.83 ± 0.15	1.07 ± 0.19
**10nM Insulin**	0.56 ± 0.2	1.29 ± 0.19
**100nM Insulin**	1.14 ± 0.18	1.18 ± 0.14
**VO-OHpic + 10 nM Insulin**	1.11 ± 0.19	1.05 ± 0.17
**VO-OHpic + 100nM Insulin**	1.14 ± 0.41	1.18 ± 0.21

Data are shown as mean fold change compared to control (± SEM) and are calculated from evaluated numbers/1000 cells from 4 independent experiments.

**Table 3:** Cytokinesis block proliferation index (CBPI) after treatment with insulin and PTEN inhibitor VO-OHpic.

Treatment Groups	CBPI
**Control**	1.95±0.008
**VO-OHpic**	1.95±0.004
**10nM Insulin**	1.94±0.011
**100nM Insulin**	1.96±0.004
**VO-OHpic + 10 nM Insulin**	1.97±0.005
**VO-OHpic + 100nM Insulin**	1.97±0.006

Date are shown as mean ± SEM from 4 independent experiments.

### 3.2 Oxidative stress and genomic damage in the liver of Pten-haplodeficient mice

**Fig 4:**
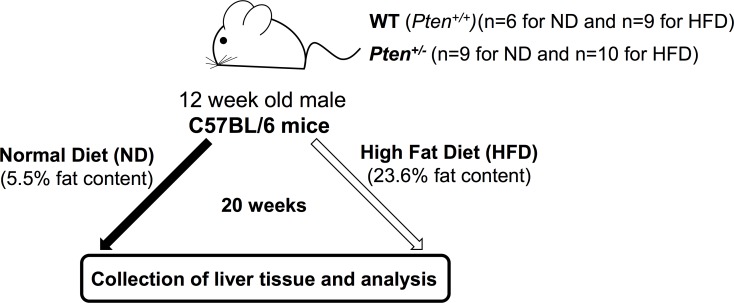
Experimental timeline. 12-week-old C57BL/6 mice (WT and Pten +/-) were fed either with ND or with HFD for 20 weeks as described in “Materials and methods”.

The overall experimental scheme is shown in **Fig 4**.

### 3.2.1 Effect of HFD on body weight, liver weight and triacylglyceride content

**Fig 5:**
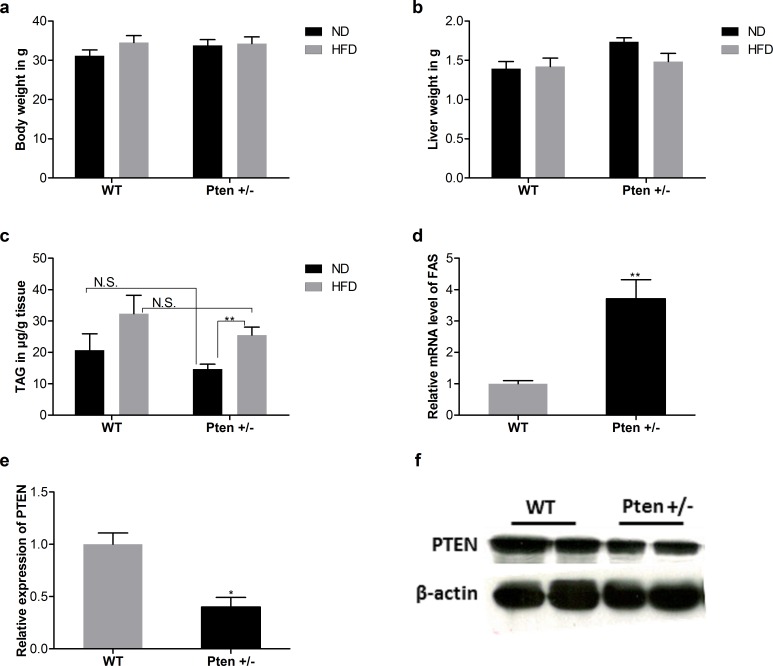
Effect of whole-body Pten haplodeficiency on metabolic parameters. a. Body weight, b. Liver weight c. Liver TAG in μg/g tissue d. Fatty acid synthase (FAS) and e. Pten expression. Mice (BW: (WT (ND, n = 6; HFD, n = 9), Pten +/- (ND, n = 9; HFD, n = 10)), Liver weight: (WT(ND, n = 6; HFD, n = 9), Pten +/- (ND, n = 9; HFD, n = 10)), TAG: (WT(ND, n = 5; HFD, n = 9), Pten +/- (ND, n = 8; HFD, n = 9)), FAS: (WT (ND, n = 6), Pten +/- (ND, n = 6)) and Pten: (WT (ND, n = 4), Pten +/- (ND, n = 4))) had been fed with ND or HFD for 20 weeks. Data are presented as mean ± SEM. * p≤0.05 and ** p≤0.01.

Pten-deficient and WT control mice were fed with ND or with HFD. No significant differences in body or liver weight were observed, but hepatic triacylglyceride (TAG) content in liver was significantly increased in Pten +/- HFD mice compared to the Pten +/- ND mice. Fatty acid synthase (FAS) was upregulated in Pten +/- mice. Pten protein expression results confirmed the Pten deficiency in this mouse model (**Fig 5**).

### 3.2.2 Dedection of intracellular ROS

**Fig 6:**
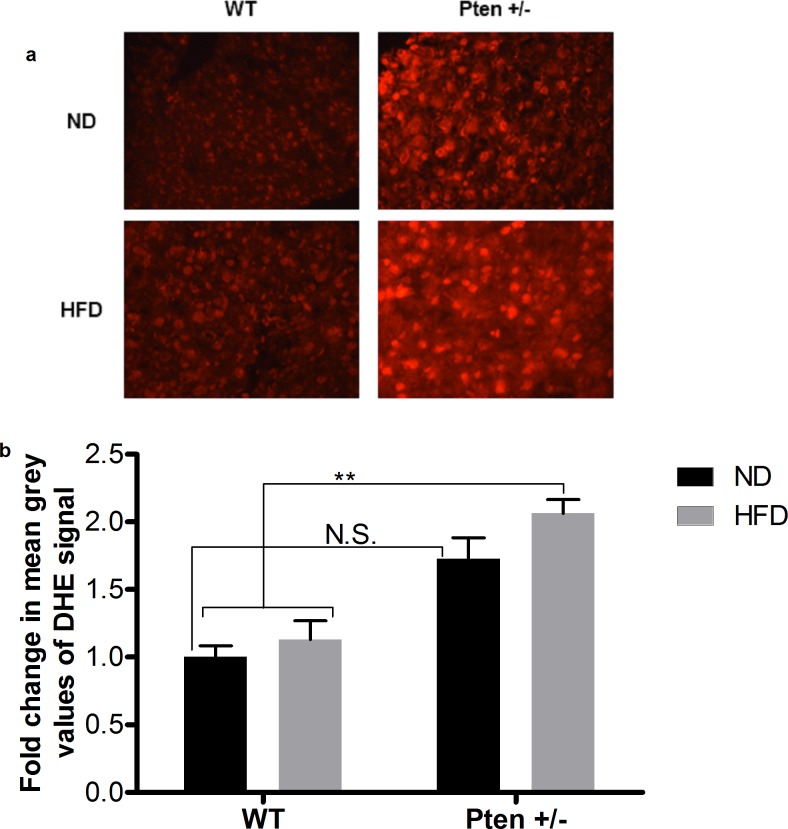
Detection of ROS in mouse liver by DHE staining. **a.** Representative pictures from DHE stained liver sections of Pten +/- and WT mice after feeding with ND or HFD. **b.** Quantification of ROS formation was achieved by measuring mean grey values of DHE signal from 200 cells using image j software. Mice (WT (ND, n = 6; HFD, n = 9), Pten +/- (ND, n = 9; HFD, n = 9)) had been fed with ND or HFD for 20 weeks. Data are presented as mean fold change compared to the WT/ND ± SEM. * p≤0.05 and ** p≤0.01.

Representative pictures of DHE stained cryosections from livers of WT and Pten +/- mice can be found in **Fig 6A**. As seen in **Fig 6B**, elevation of oxidative stress was detected after feeding with ND or with HFD in the Pten +/- group compared to the WT mice. The Pten +/- HFD group was significantly higher than the WT groups and the Pten +/- ND group.

### 3.2.3 Expression of HSP70 and HO-1

**Fig 7:**
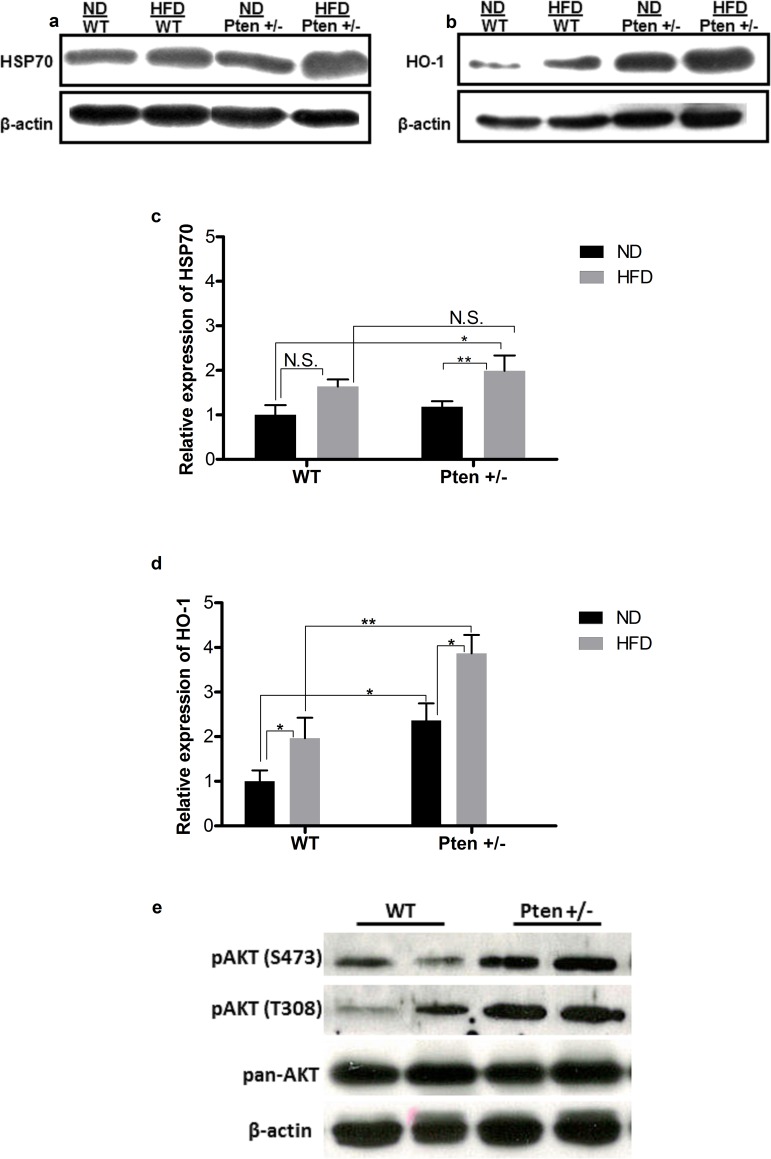
Relative expression of HSP70, HO-1, pAKT and pan-AKT in mouse liver. **a.** Representative pictures of the bands from HSP70. **b.** Representative pictures of the bands from HO-1. **c.** Quantification: The band intensity of HSP70 (WT (ND, n = 6; HFD, n = 8), Pten +/- (ND, n = 9; HFD, n = 8) was quantified by image j. Data are presented as mean fold change compared to WT/ND ± SEM. * p≤0.05 and ** p≤0.01. **d.** Quantification: The band intensity of HO-1 (WT (ND, n = 5; HFD, n = 6), Pten +/- (ND, n = 6; HFD, n = 8) was quantified by image j. Data are presented as mean fold change compared to WT/ND ± SEM. * p≤0.05 and ** p≤0.01. **e.** Representative pictures of the bands from pAKT S473, pAKT T308 and pan-AKT.

**Fig 7A** and **Fig 7B** show representative pictures of the bands from western blot analysis of HSP70 and HO-1 expression in liver. The relative quantification of the HSP70 bands can be found in **Fig 7C.** There was no difference in the expression of HSP70 protein between the WT and Pten +/- groups under ND conditions. However, a slight increase in the expression of HSP70 protein was observed in WT fed with HFD compared to the normal diet and a significant increase was observed between Pten +/- fed with HFD compared to the ND fed groups. The HSP70 expression was slightly higher in Pten +/- mice compared to the WT in HFD group. However, the difference was not statistically significant. The whole body Pten-haplodeficiency caused elevation in the expression of HSP70 protein compared to the WT mice. Quantification of the relative expression of HO-1 protein is represented in **Fig 7D**. In the ND group, there was a significant increase in the expression of HO-1 in the Pten +/- group compared to the WT group. In the HFD groups, the expression of HO-1 from mice in the Pten +/- group was elevated significantly compared to the mice in the WT group. Furthermore, a significant increase in the expression of HO-1 was observed between the mice fed with ND or HFD within the Pten +/- groups.

To assess the elevated insulin signaling due to Pten deficiency in this mouse model, western blot analysis of AKT protein was performed in ND fed group. pan-AKT and β-actin were used as loading control. Activation of PI3K/AKT signaling leads to phosphorylation of AKT protein. Western blot analysis showed an increase in AKT phosphorylation at S473 and T308 in the livers of Pten +/- mice (**Fig 7E**).

### 3.2.4 Genomic damage

**Fig 8:**
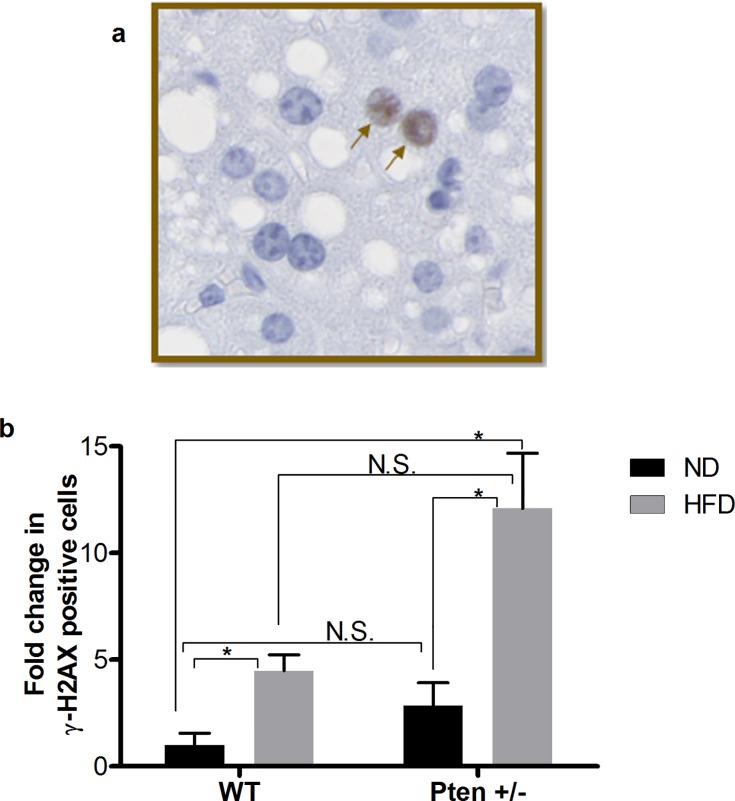
Detection of γ-H2AX on paraffin embedded mouse liver. **a.** A representative picture is shown as insert. The black arrows indicate γ-H2AX positive cells. **b.** Quantification of liver sections: 12 to 15 photos per animal were quantified by image j (WT (ND, n = 3; HFD, n = 8) and Pten +/- (ND, n = 8; HFD, n = 8). Data are presented as mean fold change of γ-H2AX positive cells compared to WT/ND ± SEM. * p≤0.05 and ** p≤0.01.

Genomic damage was monitored by an antibody staining against γ-H2AX in liver tissue. In **Fig 8A** representative picture of γ-H2AX positive cells are indicated with black arrows. In the liver of the Pten +/- mice, a slight, but non-significant elevation of DNA DSBs was observed compared to WT (**Fig 8B**). The genomic damage in the liver samples was elevated in all groups with HFD and a significant increase in the DNA damage was monitored in HFD fed mice compared to the WT mice fed with ND (**Fig 8**).

## 4 Discussion

The association of obesity, insulin resistance and T2DM to an increased incidence of cancer is well established but the underlying molecular mechanisms of carcinogenesis are still only partially understood. Hepatocyte-specific deletion of Pten, a tumor suppressor gene and negative regulator of the insulin signaling pathway, leads to an age-dependent development of hepatocellular carcinoma [16, 17]. Three hormonal mechanisms have been proposed to play a causative role for an increased cancer risk in obesity: sex hormone metabolism, insulin and insulin-like growth factor (IGF) signaling, and adipokines [4]. Particularly activation of the insulin and IGF1 receptor triggers cancer-relevant intracellular signaling cascades [25]. This study investigated the role of Pten in oxidative stress and genotoxicity *in vitro* in non-malignant liver cells (IHH) and *in vivo* in the liver of a Pten-haplodeficient mouse model. Cultured cells were used for treatment with the PTEN inhibitor VO-OHpic, and livers of whole-body Pten-haplodeficient mice were analysed.

I*n vitro*, insulin caused an elevation of oxidative stress, which was further enhanced by inhibition of PTEN. Similar observations were made regarding genomic damage induction in the comet assay, which detects DNA strand breaks and alkali-labile sites, and in the micronucleus test, with micronuclei representing a subset of chromosomal aberrations. Induction of oxidative stress and genomic damage by insulin *in vitro* were previously described by our group [26, 27] and we have identified critical steps in the mechanistic pathway [14]. In short, insulin acts through the insulin and the IGF1 receptors, activates the PI3K/AKT pathway, which then in turn induces elevated ROS production by NADPH-oxidases and mitochondria. Most likely, these ROS damage DNA or chromosomes. Proliferation-enhancing or anti-apoptotic effects of insulin were not in all cellular systems detectable, but may in certain cell types support the survival of damaged cells further. In this study, insulin especially at 10nM yielded a non-significant trend for protection from apoptosis and increased mitotic activity. Interestingly, in two different *in vitro* cellular insulin resistance models, Houstis et al. [28] showed that insulin resistance may co-exist with oxidative stress—and that an antioxidant treatment could improve the insulin resistance. This may provide a starting point for the development of protective strategies in patients with obesity and NAFLD.

PTEN inhibits the PI3K/AKT pathway by dephosphorylating PIP_3_, providing a counterpart for the stimulation of the PI3K/AKT pathway through activation of the insulin-receptor. If manipulation of PTEN activity alters the induction of insulin-induced effects, the involvement of this particular pathway in the effect is demonstrated. The *in vitro* data presented here clearly support a role of PTEN in the insulin-mediated detrimental cellular effects.

To examine a potential *in vivo* relevance, a Pten haplodeficient mouse model was investigated. In these Pten haplodeficient animals, background oxidative stress and genomic damage was elevated. Nogueira et al., [29], showed that activation of AKT causes increased intracellular ROS by elevating oxygen consumption and inhibiting ROS scavengers. Elevated background levels of oxidative stress might be due to the reduced amount of active PTEN and AKT activation in these mice. Elevated ROS might lead increase level of spontaneous DNA damage. The liver tissue of Pten haplodeficient mice showed a non-significant increase in oxidative stress under ND compared to WT, which was further enhanced by HFD feeding. While neither Pten haplodeficiency alone nor HFD alone were sufficient to cause significant elevation of oxidative stress-related markers (except for HO-1 in knockdown versus WT), the combination of these two factors resulted in a significant increase.

Expression of the analysed two proteins (HSP70, HO-1) is oxidative stress-related, but also responds to other stimuli such as infection or hyperthermia. High-energy expenditure also enhances the expression of HSP70 and vice versa [30]. HO-1 is a protective enzyme, which contributes to reduce oxidative stress and inflammatory response. Under normal conditions, HO-1 expression is low, but it can be stimulated by stress conditions like oxidative stress, elevated cytokines, growth factor and hypoxia [31, 32]. In accordance with our data, Brunt et al. [33] demonstrated an interrelationship of AKT and HO-1 expression on a translational and post-translational level. HO-1 inhibits the migration and tumor growth of human hepatocellular carcinoma cells *in vitro* [34]. HO-1 is frequently overexpressed in human HCC and down-modulation of HO-1 *in vivo* and *in vitro* resulted in increased cellular damage [35]. For extrahepatic malignancies, Alaoui-Jamali et al., [36] showed *in vivo* and *in vitro* elevated HO-1 expression in prostate cancer cells and the inhibition of HO-1 helped the reduce invasive features of the cancer cells. In our study, expression of HSP70 and HO-1 was studied as a sign for oxidative stress, but their own mechanistic role in the context of insulin and PTEN was not the focus and might be an interesting aspect for future studies.

To characterize the PI3K/AKT signalling in liver, expression of lipogenic gene FAS and phosphorylation of AKT were analysed. Results indicated elevated hepatic PI3K/AKT signalling in Pten deficient mice. We did not perform measurement for blood glucose and insulin level in this model, but it has already been shown in different studies that long term high fat diet feeding leads to impaired glucose tolerance and elevated blood insulin levels in C57BL/6 mice [37–39]. We have also supported these findings by showing an increase in AKT phosphorylation together with elevated FAS expression.

The DNA DSBs marker γ-H2AX was increased in HFD fed mice compared to the ND group. Especially the HFD fed Pten haplodeficient mice showed significant induction of DNA DSBs compared to ND. Similar to the observations regarding the oxidative stress markers, Pten haplodeficiency alone did not cause a significant increase in γ-H2AX in the ND group. HFD can stimulate hyperinsulinemia and worsen impaired glucose tolerance. Marshall et al., [40], demonstrated in a diabetes study which had been performed to investigate the interrelationship between high fat diet and T2DM in impaired glucose tolerance subjects that fat composition of the diet is a determinant factor in the development of T2DM. Vial et al., [41], investigated the effect of HFD on rat liver metabolism and ROS production. Following 8 weeks of HFD, increased mitochondrial ROS production and decreased fatty acid oxidation in rat liver was detected.

We observed no significant change in the body weight and the liver weight of the mice. Lipogenic gene FAS, a downstream target of AKT signalling, was upregulated in the livers of Pten deficient mice. It has been previously reported that liver specific Pten deficiency led to hepatic lipid accumulation due to elevated *de novo* lipogenesis in liver via AKT hyperactivation. However, whole-body Pten haplodeficient mice had reduced hepatic lipid content even with the hyperactivation of AKT [42]. In this animal model, Pten +/- mice showed an enhanced activation of PI3K/AKT signalling. This phenotypic difference between hepatocyte-specific Pten deficiency and whole-body Pten haplodeficient mice was suggested to depend on systemic interactions. The skeletal muscle insulin sensitivity has been shown to have inverse relationship to the hepatic *de novo* lipogenesis [43]. In the whole-body Pten haplodeficient mouse model, systemic interactions between the insulin sensitive tissues such as skeletal muscle might have a great impact on the hepatic PI3K/AKT signalling and lipid content. Even though there is reduced *de novo* lipogenesis in the liver of Pten haplodeficient mice, increased dietary intake due to HFD can contribute the fatty acid source in liver. HFD with the combination of hyperactivated AKT was enough to induce significant DNA-damage and oxidative stress in our experimental model.

We conclude that PTEN is in fact involved in insulin-mediated oxidative stress and genomic damage *in vitro*. The same mechanism might be conceivable as explanation for the *in vivo* observations. Overall, this further supports the hypothesis that the PI3K/AKT pathway is responsible for the damaging effects of pathophysiological levels of insulin.

Our data further support a causative role of PTEN in hepatic and extrahepatic carcinogenesis observed in obese subjects and highlights the potential of antioxidant treatment in patients with metabolic disease.

## Supporting Information

S1 Table Raw data belonging to Tables 1, 2 and 3 and Figs 1, 2, 3, 5, 6, 7 and 8. (XLSX)
